# Mushroom and Its Poison

**Published:** 1883-01

**Authors:** 


### THE COMMON MUSHROOM AND ITS POISON.

The current belief is that, while many fungi are virulently poisonous, others, including the common mushroom, are free from poison, and may be eaten in any quantity. When mushroom eaters show symptoms of poisoning, it is accordingly assumed that a blunder has been made, and noxious species taken for or with wholesome ones. The fact that an eminent English fungiologist is numbered among those who have lost their lives by the alleged mistake, would seem to throw grave doubt upon blunder theory, unless it be true, as some have held, that the edible species are mimicked by those that are poisonous so closely that the most expert is liable to misjudge them. The fear that this may be the case deters many from making any use of this savory and nourishing but treacherous vegetable.

At this season, when the fields abound with wild mushrooms, and when multitudes might find in them a cheap and enjoyable addition to the daily bill of fare if they were not afraid to eat them, it is a matter of considerable importance to have the real standing of fungi as food stuffs made clear.

According to recent investigations by Prof. Ponfick, of Breslau, the question seems to be, not how to distinguish poisonous from harmless species, but how to treat mushrooms of every sort in such a way as to remove or neutralize the poison which they all contain, with the proper precaution of using this class of food stuffs at all times with moderation.

Professor Ponfick finds that repeated washing with cold water removes most of the poison of mushrooms, and cooking, especially boiling, dissolves out the rest. The water in which mushrooms are boiled, however, is always poisonous, more so even than raw mushrooms. Experiments made upon dogs showed that if a dog ate one per cent. of its own weight of raw mushrooms it fell sick, but recovered ; one and a half per cent. produced violent illness ; and if the dog ate two per cent. of its weight, the result was always death. Of boiled mushrooms, dogs ate ten per cent. of their weight without harm. When the mushrooms were well washed with cold water, a larger quantity could be eaten raw without bad effects than was possible with those that were not washed ; but simple washing never removed the poison entirely. Dried mushrooms were found to be dangerous for twenty days, and also the water in which such mushrooms had been boiled. They were not really safe until after four months' drying.

The moral is : treat all mushrooms as poisonous ; carefully throw out the water in which they have been washed or boiled ; cook them well, and never eat them in large quantities. If men are no more susceptible than dogs are to the poison, a man can as safely gorge himself with well-boiled mushrooms as with beef or any other highly nitrogenous food. When otherwise cooked, or when the species is doubtful, a sparing use is always prudent.

The fact that all mushrooms and allied growths are more or less poisonous should be no bar to their use as food, proper care being taken in the cooking and eating. The common potato is not free from poison, and the juice of the root from which tapioca is made is a virulent poison. The latter poison is expelled by heat, and the former is in quantity too small to be harmful, as is the case with many other useful vegetables.

In preparing mushrooms for the table, safety is assured, not by looking for specific characteristics supposed to indicate harmlessness, but in considering all as poisonous and requiring judicious treatment to destroy or remove tbeir noxious qualities. This properly attended to, mushrooms and many other fungi are not only edible, but really delicious and valuable food stuffs.

